# A mixed-method study to inform the development of long-acting injectable TB treatments

**DOI:** 10.5588/ijtldopen.25.0424

**Published:** 2026-04-13

**Authors:** A.J.F. Bayot, M.T. Gler, E.E. Tolley, L. Lorenzetti, E. Namey, N. Dinh, A. Martinez, C. Audibert, S. Foraida, J. Osborn, D. Holtzman, C. Vinnard, E.A. Talbot, C. Wells

**Affiliations:** 1 TB HIV Innovations and Clinical Research Foundation, Silang, Philippines;; 2 FHI 360, Global Health and Population Research, Durham, NC, USA;; 3 MMV Medicines for Malaria Venture, Geneva, Switzerland;; 4 TB Alliance Research and Development, New York City, NY, USA;; 5 Gates Medical Research Institute, Cambridge, MA, USA;; 6 Geisel School of Medicine at Dartmouth, Hanover, NH, USA.

**Keywords:** tuberculosis, the Philippines, treatment adherence, target regimen profiles

## Abstract

**BACKGROUND:**

Development of long-acting injectable (LAI) formulations of TB drugs could transform global TB management. We assessed three hypothetical LAI treatment scenarios and seven product-specific characteristics among TB patients and survivors, TB clinicians, and other key stakeholders.

**METHODS:**

A two-stage, mixed-method study was conducted in the Philippines. Stage 1 included semi-structured interviews with 15 key informants (KIs) from public health organisations, government, and clinical practices. Stage 2 entailed face-to-face surveys and a discrete choice experiment (DCE) with TB health providers (n = 51), adult TB patients on treatment (n = 247), and TB survivors (n = 54) in one urban and one semi-urban setting.

**RESULTS:**

Common themes identified through KI interviews included goals of minimising treatment burden, maximising adherence and efficacy, primary care setting preference, and the ability to monitor response. Relative to the oral standard of care, most participants reported that the LAI TB treatment scenarios were acceptable. Body placement of the injection was a driving characteristic of treatment choice for patients; preference for LAI treatment delivered through a primary care setting was reported as positively influencing treatment choice across respondent types.

**CONCLUSION:**

Multi-setting stakeholder input should guide development of LAI TB treatment so that developers can aim for acceptable product characteristics.

Growing rates of drug resistance and continued barriers to patient care have contributed to the call for more effective and user-friendly TB treatment options,^1–3^ reflected in the recent update to the WHO target regimen profiles (TRPs) for TB treatment.^4^ Long-acting injectable (LAI) forms of TB treatment could significantly reduce TB-related morbidity and mortality if they improve treatment uptake, adherence, and regimen completion, and could lessen the socio-economic burden on TB patients and their families. With fewer patient encounters, LAI forms of TB treatment could also reduce the burden for TB care providers. With the promise of novel TB treatment options comes a need to understand how these products would be perceived by end-users, including patients, health care providers, and health officials, in different settings and contexts. Development of these products must be guided by social and behavioural research on product trade-offs and preferences to ensure end-users’ concerns and needs are addressed before products are developed and introduced.^5,6^

We sought to identify the perceived trade-offs between potential TRP indicators of an LAI injectable TB treatment regimen among policy makers and programme implementers with expertise in TB treatment and control. We also sought to assess the acceptability of and preferences for potential LAI TB treatments among TB patients on treatment and TB survivors, including how trade-offs between potential regimens might affect treatment uptake, adherence, and persistence. Our third objective was to assess the acceptability of and preferences for potential LAI TB treatments among TB care providers.

## METHODS

We conducted a two-stage, mixed-method study at two settings in the Philippines. Stage 1 included key informant (KI) consultations with 15 national and regional TB experts, programme managers, and others. Stage 2 was comprised of tablet-based, face-to-face surveys with adult TB patients on treatment, TB survivors, and TB health care providers.

### Study setting

This study was conducted in the Philippines, which has a high TB burden (fourth highest globally) and low TB notification rates,^7,8^ at one urban and one adjacent semi-urban setting, metropolitan Manila and Cavite, respectively. As of 2022, the National Capital Region (where Manila is located) saw 53,837 TB cases among a population of 12.9 million (incidence of 417/100,000 population).^9^ Cavite is the second smallest province in the Calabarzon region. In 2022, there were 18,408 TB cases reported in Cavite (incidence of 424/100,000 population).^9^

### Procedures

*Stage 1*: We conducted interviews with 15 stakeholders, including national, regional, and community-based TB advisors who have knowledge of and responsibilities for policy and programme decisions related to TB treatment in the Philippines. We then refined a set of LAI TB treatment scenarios that were integrated into Stage 2 surveys with patients, survivors, and providers (Box 1). Scenarios were selected to evaluate a range of characteristics such as the number of health care encounters, the number of injections, and the requirement for an oral medication lead-in period. We crafted the scenarios to align with the development pipeline, such that the scenarios were thought to be technically plausible at the time of this writing.

Box 1. Descriptions of each LAI TB scenario and current standard of care treatment shared with key informants and survey respondents in the Philippines.

Standard of care treatment for drug-susceptible TB (DS-TB) patients and survivors: The current standard of care to treat people with DS-TB involves having them take three to five pills by mouth daily. It is recommended that patients take their pill while being observed – either in person (DOT) or by video (VOT) – by a health care worker. However, given the challenges of daily DOT, patients generally self-administer their pills at home.

Standard-of-care treatment for multidrug-resistant TB (MDR-TB) patients and survivors: For treating drug-resistant forms of TB (including MDR-TB and extensively drug-resistant TB), the patient takes a 6-month daily oral regimen of BPaLM or BPaL. The patient takes a combination of seven pills (tabs) daily and needs to return to the clinic/local health centre every 2 weeks to obtain a refill of their medications.

LAI TB Treatment Scenario 1: Now, I want to present you with a new scenario for a potential future treatment. In this scenario, a one-time TB treatment might be getting 3–4 injections at a single visit. Each shot would contain a different TB drug. The shot would be given deep in the muscle tissue in the buttocks. Patients may experience more or stronger side effects, like injection site pain, headache, or nausea. Pain medication could be recommended when receiving this treatment. This treatment would likely be delivered at a second or third tier hospital.

LAI TB Treatment Scenario 2: Now, I want to present you with a treatment that involves getting two injections once a month over 4 months. Pain would be minimal, but some side effects could be possible. The injections would contain two highly potent but new drugs. The injections for this new treatment would be delivered just under the skin in the upper arm. Because the injection is just under the skin, it may leave a small bump. The bump will go away on its own, but it may take several days or weeks. Patients could receive their injection at their primary care clinic.

LAI TB Treatment Scenario 3: Now, I want to present you with a long-acting TB treatment option with injections given every 3 months. This treatment may not be an option for patients who are using some HIV treatment or prevention drugs. The injection may cause side effects, like injection site swelling and low fever. Because the drug is new, the patient must first take the drug in pill form for 1 month. If the patient can tolerate the drug, she/he will receive an injection in the upper arm muscle. The patient will receive one more injection 3 months after the first injection. The patient could obtain the injections at a local primary care clinic or specialty clinic.

*Stage 2*: Within each setting, we recruited participants from various tiers of health facilities and clinical settings where TB patients are treated. In Stage 2, we aimed to recruit 25 providers, 125 TB patients, and 25 TB survivors per setting, totalling 350 participants across Manila and Cavite. Across all health facility tiers, we sampled facilities based on logistical considerations and where existing relationships facilitated data collection. Once health facilities were selected within each stratum, we conducted convenience sampling of health care providers, TB patients, and TB survivors separately. Health care providers included physicians, nurses, counsellors, or community-based staff involved in screening, diagnosis, and/or treatment of TB. Health care providers reviewed the same scenarios as TB patients on treatment and TB survivors but were asked to reflect on acceptability of the scenarios, reflecting service delivery and provider considerations. From these venues, we also identified adult TB patients, defined as individuals currently receiving treatment for active drug-susceptible or drug-resistant TB disease, and adult TB survivors, defined as individuals having completed a course of TB treatment within the previous 24 months. Stage 2 tablet-based surveys were conducted face-to-face by a local field team, comprised of trained research staff. We collected data on patient demographics and perceptions of acceptability related to LAI treatment scenarios relative to the current standard of care (SOC) treatment, as shown in Box 1. The interviewer asked about the acceptability of each of these scenarios relative to the base scenario, which is SOC treatment with all-oral medications. The Stage 2 survey included a discrete choice experiment (DCEs) to assess trade-offs between characteristics related to different treatment scenarios. DCEs are a type of experimental design that was developed in the field of economics to predict choice at a population level between a limited number of options (in this case, potential LAI TB treatments) that are characterised by different features.^10^ The DCE included key product characteristics and related response options that defined alternative LAI TB treatment scenarios (Table 1), with additional variations beyond the three scenarios shown in Box 1. The DCE attributes are slightly different from Stage 1 scenarios based on participant feedback received during pilot testing.

**Table 1.** Product characteristics and options for long-acting injectable TB treatment.

Characteristic	Options		
Number of injections (per visit)	One injection	Two injections	Three injections
Duration	Every month for 4 months	Every 2 months for 8 months	One time only
Placement of shots on body	Buttock	Thigh	Upper arm
Oral therapy needed	No oral therapy, immediately receive injection	Two weeks of oral therapy, then receive injection	Two months of oral therapy, then receive injection
Injection site pain	Mild, does not require analgesic medication	Moderate, may require analgesic medication	Severe, requires analgesic medication
Side effects	Headache	Swelling	Rash
Service delivery system	Community health posts	Hospitals	Community-based organisations

### Data analysis

In Stage 1, all transcripts were imported into NVivo 14 (Lumivero, Denver, CO) for structural and thematic coding, and then coded material was summarised and synthesised. Survey data in Stage 2 were descriptively summarised using tabular formats, separately for each setting. The DCE data were analysed with regression modelling in a random utility framework, which assumes that individuals consider trade-offs when making decisions and choose the option that offers the greatest utility, determined by how much importance they place on the characteristics associated with the product. Specifically, we used mixed logit regression models, an approach commonly used to analyse discrete choice data as it allows attribute coefficients to vary across respondents, accounting for preference heterogeneity. For each specific response option within a given characteristic, the model coefficient provided an estimate of preference relative to the mean effect for the characteristic, with an associated confidence interval. Thus, generalisation is limited to the specific response options for each characteristic and does not extend to other options not explicitly defined. Data analysis was conducted in STATA and R.

### Ethical statement

This study received approval from the Makati Medical Center Institutional Review Board (based in the Philippines), and exempt determination from the Protection of Human Subjects Committee at FHI 360 (Durham, NC).

## RESULTS

### Stage 1

In general, KIs reported that new LAI TB treatment regimens would be welcome in the Philippines, provided that strong evidence supports the safety and efficacy of any new treatments, and that WHO endorsement and stringent regulatory authority approvals are obtained. Nevertheless, KIs anticipated barriers to the introduction and adoption of new treatment regimens, including logistical challenges related to cost, supply chain, and distribution, as well as concerns regarding patient discomfort with injections. KIs concluded it would be important to assess the capacity of the health care system, especially outside larger cities and/or hospitals to ensure appropriate provider training, service delivery systems for patient follow-up, and supply chain management (Box 2).

Box 2. Summary of themes and selected verbatims from key informant interviews.

**Opportunities for LAI regimens:**

“Most [patients] would prefer less frequent drug administration. So, for the injectable, it will require fewer visits, less time per visit for the patients and also less time … to administer the drugs. And they will not take it every day. So that's a good opportunity for them.”

**Logistical issues related to the health care system:**

“…The availability of injectable TB drugs will be foremost because, for it to work, you have to have the drug readily available. … and of course, can it be injected in the primary health center like what we have in our city health offices? Or will it be needing a much higher [tier], like a secondary or tertiary hospital for it to be given?…We have to know all of those things, to see if it's feasible in our country.”

**Feedback on illustrative LAI TB scenarios**

*Scenario 1: Four injections in a one-time treatment:*

“I think maybe 98% of [patients] would take it because this is what we usually call ‘one-time big-time’. You know, you get to be treated for TB in one day, and that's it – you’re cured. I will have to choose this, everybody will choose this (laughs).”

“[Scenario 1] is not patient friendly – deep IM with different medications meaning 4 times the pain in a day.”

*Scenario 2: Two injections monthly for 4 months:*

“…[This scenario is] shorter compared to the standard 6-month treatment regimen. Fewer injections…are less burdensome than the four injections in Scenario 1. And the ability to administer the treatment at primary care clinics improves accessibility for a wider range of patients, and [there would be] minimal pain [with] subcutaneous injections.”

*Scenario 3: 1 month daily oral run-in followed by two injections 3 months apart:*

“… as a health care provider, it's quite confusing. I will start my patient with a monthly daily oral and then I will shift that patient into monthly injections, and then there are other considerations that if the patient can tolerate the drug and so on and so forth. I think comparing it with the previous two scenarios, this is more confusing.”

### Stage 2

#### Participant characteristics and medical history

Surveys were administered to n = 352 participants, including 247 TB patients actively on treatment (70.2% of respondents), 54 TB survivors (15.3% of respondents), and 51 health care providers (14.5% of respondents) in Manila and Cavite, with the sample split evenly across settings. The characteristics of patients and survivors are shown in Table 2. TB patient and TB survivor survey respondents were 44.9% women, with a history of multidrug-resistant TB (MDR-TB) in 27.2% of respondents. Overall, 18.9% reported personal experience with injectable medication. Most providers had six or more years of experience with TB health care delivery, and included medical doctors, nurses, clinical officers, and community health workers (Table 3).

**Table 2.** Characteristics of patients, including TB patients and TB survivors, disaggregated by location.

	Manila (urban)	Cavite (semi-urban)	Total
	N = 150	N = 151	N = 301
Age			
Mean (SD)	43.56 (13.72)	41.94 (15.50)	42.75 (14.64)
Sex at birth			
Male	85 (56.7%)	81 (53.6%)	166 (55.1%)
Female	65 (43.3%)	70 (46.4%)	135 (44.9%)
Highest level of education completed			
Never been to school	1 (0.7%)	1 (0.7%)	2 (0.7%)
Early childhood education programme	0 (0.0%)	2 (1.3%)	2 (0.7%)
Primary school	25 (16.7%)	24 (15.9%)	49 (16.3%)
Secondary school	72 (48.0%)	76 (50.3%)	148 (49.2%)
University or higher	39 (26.0%)	39 (25.8%)	78 (25.9%)
Technical school	13 (8.7%)	9 (6.0%)	22 (7.3%)
Anyone in the household diagnosed with TB in the past 1 year			
No	104 (69.3%)	36 (23.8%)	140 (46.5%)
Yes	46 (30.7%)	115 (76.2%)	161 (53.5%)
Ever diagnosed with drug-resistant TB	34 (22.7%)	48 (31.8%)	82 (27.2%)
HIV status^A^			
Negative	72 (48.0%)	110 (72.8%)	182 (60.5%)
Positive	1 (0.7%)	10 (6.6%)	11 (3.7%)
Unknown	75 (50.0%)	31 (20.5%)	106 (35.2%)
Prefer not to say	2 (1.3%)	0 (0.0%)	2 (0.7%)
If positive, taking ART			
Yes on ART for <6 months	1 (100.0%)	6 (60.0%)	7 (63.6%)
Yes on ART for >6 months	0 (0.0%)	4 (40.0%)	4 (36.4%)
Ever taken medications by injection instead of taking them by mouth	18 (12.0%)	39 (25.8%)	57 (18.9%)

ART = antiretroviral therapy.

A By self-report.

**Table 3.** Characteristics of providers, disaggregated by location.

	Manila (urban)	Cavite (semi-urban)	Total
	N = 27	N = 24	N = 51
Sex at birth			
Male	4 (14.8%)	3 (12.5%)	7 (13.7%)
Female	23 (85.2%)	21 (87.5%)	44 (86.3%)
Highest level of education completed			
Secondary school	2 (7.4%)	3 (12.5%)	5 (9.8%)
University or higher	25 (92.6%)	19 (79.2%)	44 (86.3%)
Technical school	0 (0.0%)	2 (8.3%)	2 (3.9%)
Professional background			
Medical doctors or medical officers	15 (55.6%)	3 (12.5%)	18 (35.3%)
Nurses	11 (40.7%)	8 (33.3%)	19 (37.3%)
Clinical officer/medical licentiate	1 (3.7%)	6 (25.0%)	7 (13.7%)
Community health worker	1 (3.7%)	8 (33.3%)	9 (17.6%)
Type of facility			
Specialty hospitals	6 (22.2%)	3 (12.5%)	9 (17.6%)
Teaching hospitals	2 (7.4%)	3 (12.5%)	5 (9.8%)
Public primary health centres	13 (48.1%)	15 (62.5%)	28 (54.9%)
Private–public mix DOT	1 (3.7%)	1 (4.2%)	2 (3.9%)
Private offices/clinics	5 (18.5%)	2 (8.3%)	7 (13.7%)
Year of experience in managing treatment and prevention for TB patients			
0–5	9 (33.3%)	8 (33.3%)	17 (33.3%)
6–10	8 (29.6%)	10 (41.7%)	18 (35.3%)
11–15	6 (22.2%)	2 (8.3%)	8 (15.7%)
16–20	1 (3.7%)	3 (12.5%)	4 (7.8%)
21 or more	3 (11.1%)	1 (4.2%)	4 (7.8%)
Number of patients for whom TB prevention or treatment was prescribed in last year			
<10	1 (3.7%)	3 (12.5%)	4 (7.8%)
11–100	7 (25.9%)	10 (41.7%)	17 (33.3%)
101–500	15 (55.6%)	7 (29.2%)	22 (43.1%)
>500	4 (14.8%)	4 (16.7%)	8 (15.7%)

DOT = directly observed treatment.

#### Acceptability of LAI TB treatment among TB patients on treatment, TB survivors, and providers

**Figure 1.**
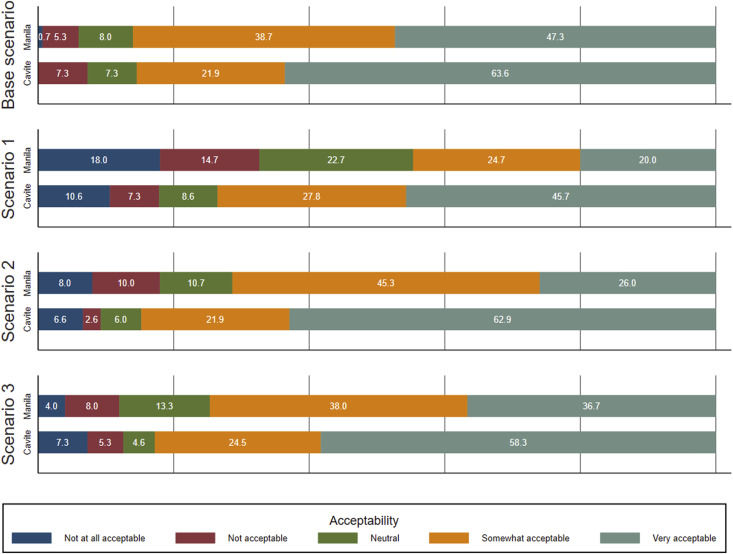
Bar chart comparing acceptability of scenarios by TB patients and TB survivors, by location. Scenario 1 corresponds to four injections in a one-time treatment. Scenario 2 corresponds to two injections monthly for four months. Scenario 3 corresponds to 1 month daily oral run-in followed by two injections 3 months apart. Acceptability of each of these scenarios is relative to the base scenario (standard of care).

When considering acceptability of the SOC treatment, most TB patients and TB survivors found this treatment to be either ‘very acceptable’ (55.5%) or ‘somewhat acceptable’ (30.2%), representing 85.7% of participants (Figure 1). For the 6.9% of participants reporting the SOC treatment was ‘not acceptable’ or ‘not at all acceptable’, approximately one half cited side effects as making the treatment unacceptable, while others noted a decreasing motivation to use the treatment over time. For each LAI TB treatment scenario (Box 1), TB patients and TB survivors were asked to compare the acceptability of this treatment to the SOC treatment. Most participants rated each of the three LAI TB treatment scenarios as acceptable relative to the SOC treatment. Providers consistently rated the acceptability of Scenario 2 and Scenario 3 higher than for Scenario 1 (data not shown). However, some patients and survivors also reported Scenarios 1 (25.3%), 2 (13.6%), and 3 (12.2%) to be ‘not acceptable’ or ‘not at all acceptable’ relative to SOC treatment. When examined by TB type, we observed that overall acceptability (either somewhat or very acceptable) among MDR-TB participants was 68.2% for Scenario 1 relative to the SOC, compared with 55.7% among drug-susceptible TB (DS-TB) participants. For Scenario 2, overall acceptability was 84.1% (MDR-TB) versus 75.8% (DS-TB). For Scenario 3, overall acceptability was 79.3% (MDR-TB) versus 78.5% (DS-TB). By location, respondents from Cavite (semi-urban) were consistently more likely to rank an attribute as ‘very’ acceptable compared with respondents in Manila (urban) who typically erred on the side of ‘somewhat’ acceptable, rather than neutral or unacceptable.

#### Discrete choice experiment with LAI TB treatment scenarios and characteristics

**Figure 2.**
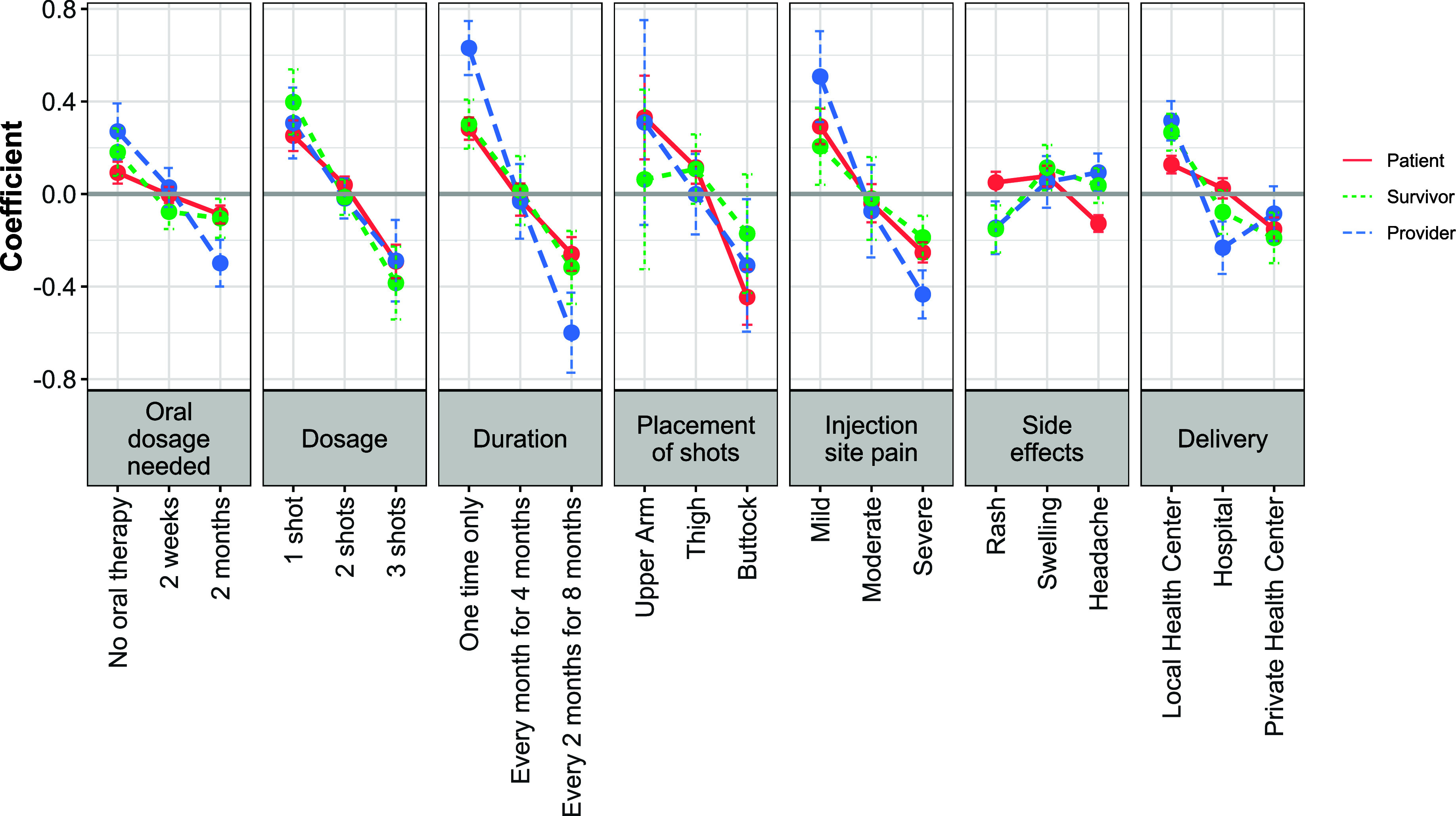
Preference for long-acting injectable (LAI) TB characteristics, by respondent group. For each level of a characteristic, the coefficient demonstrates the influence on either accepting (positive) or rejecting (negative) a treatment choice. The coefficient for each option within a characteristic was determined separately for active TB patients, TB survivors, and TB care providers.

DCE results were disaggregated by stakeholder groups, namely active TB patients, TB survivors, and health care providers. Figure 2 provides an overview of preference results by each group. Across the seven characteristics, placement of the injection on the body was most important for TB patients, while the need for an oral lead-in was least important. Among TB survivors, per visit dosage was most important, with side effects being least important. Providers rated duration/spacing between injections as most important, compared with side effects as being least important. An active TB patient’s decision to choose an LAI treatment scenario was significantly influenced by the following characteristics and response options: having mild injection site pain that does not require medication (0.29, *P* < 0.001), receiving a one-time only injection (0.28, *P* < 0.001), receiving one injection per visit (0.25, *P* < 0.001), and receiving treatment at a local health centre (0.13, *P* = 0.001). In contrast, an active TB patient’s decision to reject an LAI treatment scenario was influenced by the following options and characteristics: receiving an injection in the buttocks (−0.45, *P* < 0.001), receiving three injections per visit (−0.29, *P* < 0.001), receiving an injection every 2 months for 8 months (−0.26, *P* < 0.001), having injection site pain that is severe and requires analgesic medication (−0.25, *P* < 0.001), receiving treatment at a private health centre (−0.15, *P* = 0.003), and having a headache as a side effect (−0.13, *P* < 0.001).

Among TB survivors, there were three response options that significantly influenced their decision to choose an LAI treatment scenario: receiving one injection per visit (0.40, *P* = 0.005), receiving a one-time only injection (0.30, *P* = 0.004), and getting services from a local health centre (0.27, *P* = 0.001). The following three characteristics and options significantly influenced their decision to reject an LAI treatment scenario: needing three injections per visit (−0.38, *P* = 0.015), receiving injections every 2 months for 8 months (−0.32, *P* = 0.044), and having severe injection site pain that requires analgesic medication (−0.19, *P* = 0.045).

Regarding providers, their decision to choose an LAI treatment scenario was influenced by the following characteristics and response options: providing a one-time only injection (0.63, *P* < 0.001), an injection with mild injection site pain (0.51, *P* = 0.01), delivering the injection at a local health centre (0.32, *P* < 0.001), providing one injection per visit (0.31, *P* = 0.045), and not requiring an oral lead-in (0.27, *P* = 0.026). Four characteristics significantly affected their decision to reject an LAI treatment option: providing injections every 2 months over an 8-month period (−0.60, *P* = 0.001), providing an injection that causes severe injection site pain and requires analgesic medication (−0.43, *P*=<0.001), requiring patients to take a 2-month oral lead-in prior to the first injection (−0.30, *P* = 0.003), and providing treatment at a hospital (−0.23, *P* = 0.041).

## DISCUSSION

The goal of this work was to inform early-stage development of a novel LAI TB treatment regimen, as guided by the perceived preferences, trade-offs, barriers, and needs of KIs in the TB programme, end-users such as TB health care providers, and persons taking or having completed TB treatment. Overall, we found interest in an LAI TB treatment option across all groups.

KIs and TB health care providers viewed LAI treatment options as potentially reducing barriers to adherence posed by daily oral therapy. Given that an LAI TB treatment might include a novel agent, the possibility of including an oral lead-in could be an acceptable approach, based on the responses of active TB patients and TB survivors. Although a shorter treatment duration may be the ultimate goal among product developers and providers, as this would reduce adherence-related burden, this was not necessarily prioritised over the number of injections per visit. Instead, allowing patients to build familiarity with an injectable and ensure a less painful experience seemed to be more important than a significant reduction in treatment duration. Contextually, the preferences and concerns with regard to LAI treatment of latent TB infection, known as TB preventive therapy, was investigated in a recent survey of patients and health care providers in India and South Africa, with 75% of respondents expressing a strong willingness to try an LAI treatment for latent TB infection.^11^ A survey of patients and providers to understand preferences around long-acting malaria chemoprevention, conducted in Kenya and Zambia, found high levels of enthusiasm in both groups of respondents.^12^

High levels of interest in LAI treatment notwithstanding, we also found that the current SOC treatment was viewed favourably by survey respondents at both sites. A smaller but noteworthy proportion of respondents indicating an LAI treatment scenario would not be acceptable over daily oral therapy suggests that an LAI option may not appeal to all TB patients.

One limitation was that a single country setting was used to assess stakeholder preferences, and the composition of respondents may not be representative of the national level. Although the recruitment strategy included four levels of health facilities across two settings, there is potential for selection bias from convenience sampling within each setting and tier. Additionally, the respondents’ perceptions and estimations that were captured by the field survey may be influenced by recall bias, as their answers reflect their views of their practice and/or experience. The regional variation in acceptability of an LAI option relative to the SOC suggests a need to explore whether differences are driven by social desirability bias or potentially a lack of choice in treatment options for participants in more rural areas.

## CONCLUSION

Our results indicate broad interest in LAI TB treatment, despite concerns over injection site pain and the potential need for delivering multiple injections at a single visit. Efforts to support the development of LAI TB treatment should continue to be guided by stakeholder input to understand acceptable product characteristics.
